# Supplementary material for: Single Cell Transcriptomic and Chromatin Profiles Suggest Layer Vb Is the Only Layer With Shared Excitatory Cell Types in the Medial and Lateral Entorhinal Cortex
Source: Front Neural Circuits. 2022 Jan 26;15:806154. doi: 10.3389/fncir.2021.806154 (PMC8826650; doi:10.3389/fncir.2021.806154)
Supplement: Supplementary file 14 [file Data_Sheet_1.ZIP › Sup_vs_Deep_Motifs/homerResults/motif1.similar.html]

motif1

## Information for motif1

G
A
C
T
T
A
C
G
T
G
A
C
T
C
G
A
C
A
T
G
T
A
C
G
C
A
G
T
C
T
A
G
A
G
T
C
C
G
T
A
  
Reverse Opposite:  

G
C
A
T
T
C
A
G
G
A
T
C
G
T
C
A
A
T
G
C
G
T
A
C
A
G
C
T
A
C
T
G
A
T
G
C
C
T
G
A
  

|  |  |
| --- | --- |
| p-value: | 1e-3125 |
| log p-value: | -7.197e+03 |
| Information Content per bp: | 1.650 |
| Number of Target Sequences with motif | 13582.0 |
| Percentage of Target Sequences with motif | 40.53% |
| Number of Background Sequences with motif | 1183.6 |
| Percentage of Background Sequences with motif | 13.86% |
| Average Position of motif in Targets | 274.9 +/- 224.2bp |
| Average Position of motif in Background | 396.2 +/- 323.3bp |
| Strand Bias (log2 ratio + to - strand density) | 0.1 |
| Multiplicity (# of sites on avg that occur together) | 1.32 |
| Motif File: | file (matrix) reverse opposite |

### Similar de novo motifs found

|  |  |  |  |  |  |  |  |
| --- | --- | --- | --- | --- | --- | --- | --- |
| Rank | Match Score | Redundant Motif | P-value | log P-value | % of Targets | % of Background | Motif file |
| 1 | 0.966 | C T G A T G C A T C G A A G T C C G T A A G T C A G T C A C G T A C T G A G C T A T C G G C A T | 1e-3086 | -7106.753265 | 42.51% | 15.29% | motif file (matrix) |
| 2 | 0.982 | G A C T T G C A A G T C C G T A A C T G A C T G A C G T A C T G | 1e-2906 | -6693.442974 | 44.28% | 17.12% | motif file (matrix) |
| 3 | 0.948 | A G T C C T G A A G T C A T G C A C G T C T A G T G A C C T G A | 1e-1456 | -3354.524879 | 31.09% | 13.69% | motif file (matrix) |
| 4 | 0.874 | A G T C G T A C G C T A A G T C A G T C C G A T C T A G A C G T G T A C G T A C | 1e-593 | -1366.850255 | 50.43% | 36.44% | motif file (matrix) |
| 5 | 0.735 | A G T C C G T A A C T G A C T G A C G T A C T G A G T C C G T A A C T G A C T G A C G T A C T G | 1e-315 | -725.648561 | 12.10% | 6.44% | motif file (matrix) |
| 6 | 0.714 | A T G C A G T C C G T A A C G T A G T C A C G T A C T G A G C T | 1e-266 | -613.405887 | 27.68% | 19.76% | motif file (matrix) |
| 7 | 0.774 | T C A G A G T C C G T A C A T G G T A C A C G T A C T G A G T C G A C T A T G C | 1e-219 | -506.393914 | 49.78% | 41.19% | motif file (matrix) |
| 8 | 0.693 | C G T A C G T A T C G A T C G A A G T C C G T A C T A G C G T A C G A T A C T G A T C G G C A T | 1e-213 | -490.887945 | 24.30% | 17.53% | motif file (matrix) |
| 9 | 0.777 | T G C A A T C G T G A C C G T A C T A G C T A G A C G T A C T G C G T A A C T G T A G C G C T A | 1e-161 | -370.926798 | 0.58% | 0.04% | motif file (matrix) |
| 10 | 0.745 | T G A C G T C A T A C G C T A G A C G T C G T A C T G A T G A C | 1e-144 | -332.930698 | 25.76% | 19.98% | motif file (matrix) |
| 11 | 0.751 | C G T A A G T C C G T A A G T C A G T C A C G T A C T G C G T A A C T G C G A T | 1e-109 | -252.594673 | 0.63% | 0.09% | motif file (matrix) |
| 12 | 0.781 | G A T C C T G A A C T G A T G C A G C T C T A G A G T C A C T G | 1e-104 | -241.118547 | 30.70% | 25.42% | motif file (matrix) |
| 13 | 0.738 | T C G A C A T G A C G T T G A C C G T A A C T G A C T G C G A T T A C G A G C T | 1e-102 | -235.228931 | 7.83% | 5.06% | motif file (matrix) |
| 14 | 0.761 | A C T G C G T A C G T A A G T C C G T A A G T C A G T C A C G T A C T G C G T A | 1e-83 | -191.581607 | 0.33% | 0.02% | motif file (matrix) |
| 15 | 0.642 | T A C G C G T A A T C G C G A T T G A C G T A C G C T A A T G C G T A C G C A T A T C G C T G A | 1e-59 | -138.110617 | 0.27% | 0.03% | motif file (matrix) |
| 16 | 0.612 | A C T G C G T A A G T C C G T A A C G T G T A C C G A T A C T G | 1e-45 | -105.789889 | 9.23% | 7.14% | motif file (matrix) |
| 17 | 0.610 | A C G T A C T G A G T C A C G T A G T C A G T C A C G T A C T G A G T C A C T G A C G T A G T C | 1e-32 | -75.599387 | 0.31% | 0.07% | motif file (matrix) |
| 18 | 0.712 | A C G T C G T A A G T C A G T C A C G T A C T G A G T C A C G T | 1e-24 | -56.614896 | 2.80% | 1.97% | motif file (matrix) |
